# A nonconforming scheme for non-Fickian flow in porous media

**DOI:** 10.1186/s13660-017-1419-7

**Published:** 2017-06-19

**Authors:** Peizhen Wang, Liying Jiang, Shaochun Chen

**Affiliations:** 1 School of Mathematics and Statistics, North China University of Water Resources and Electric Power, Zhengzhou, 450011 China; 2 Department of Basic Science, Zhengzhou Vocational College of Finance and Taxation, Zhengzhou, 450048 China; 3 School of Mathematics and Statistics, Zhengzhou University, Zhengzhou, 450052 China

**Keywords:** non-Fickian flow, interior penalty method, Wilson nonconforming element, convergence analysis

## Abstract

In this paper, we construct a semi-discrete scheme and a fully discrete scheme using the Wilson nonconforming element for the parabolic integro-differential equation arising in modeling the non-Fickian flow in porous media by the interior penalty method. Without using the conventional elliptic projection, which was an indispensable tool in the convergence analysis of finite element methods in previous literature, we get an optimal error estimate which is only determined by the interpolation error. Finally, we give some numerical experiments to show the efficiency of the method.

## Introduction

Consider the numerical solution of the non-Fickian flow in porous media modeled by an initial boundary value problem of the following parabolic integro-differential equation:

1.1 $$ \textstyle\begin{cases}\phi u_{t}- \operatorname{div} (A\nabla u+\int_{0}^{t} B(s)\nabla u(x,s)\,ds )=f,&\text{in }\Omega\times(0,T],\\ u=0,& \text{on }\partial\Omega\times(0,T],\\ u(x,0)=u_{0}(x),&\forall x\in\Omega. \end{cases} $$

This kind of flow is complicated by the history effect, which characterizes various mixing length growths of flow. This model of equation is widely applied in many fields, such as in non-Fourier models for heat conduction in materials with memory, in engineering models for nonlocal reactive transport in porous media and in the theory of nuclear reactors. There are many studies on the existence and uniqueness of its solution, also, on the numerical solution of it.

There are many papers on the numerical methods for this kind of problems. Ewing et al. [1] derived the finite volume methods, and Jiang [2] considered the mixed element methods when $A,B$ are proportional to a unit matrix for this problem. Ewing et al. [3] and [4] presented the $L^{2}$-error estimate and $L^{\infty}$-error estimate of the mixed element methods for this problem in a general case. The mixed element method can obtain the approximations of *u* and *σ* simultaneously, but it needs the Ladyzhenskaya-Babuska-Brezzi (LBB) consistency condition. To overcome this disadvantage of mixed element methods, Rui [5] gave some split least-squares finite element procedures and the convergence analysis with optimal accuracy. Besides these methods, Cui Xia [6] presented an A.D.I. Galerkin method, and Cannon and Lin [7] considered the finite element methods for this problem by use of the generalized elliptic projection. When using the conforming finite element methods approximation of this problem, it can lead to too much degree of freedom. For the nonconforming finite element methods, there are two disadvantages: first, it needs analyze the consistency term; secondly, the convergence order is not optimal since the order of the interpolation error is higher than that of the consistent error for some elements, such as the Wilson element for the second-order problems [8] and the Adini element for the forth-order problems (see [9]).

To overcome these disadvantages of the finite element methods, the interior penalty method was introduced. The study of this method traces back to the 1970s. Douglas etc. provided a framework for the analysis of a large class of discontinuous methods for second-order elliptic problems in [10] and a semi-discrete finite element procedure for the second-order parabolic initial boundary value problem in [11]. Andreas et al. [12] analyzed the discontinuous Galerkin method for the linear second-order elliptic problem on a compact smooth connected and oriented surface in $R^{3}$. For a fourth-order elliptic boundary value problem, Engel et al. [13] proposed an interior penalty method that uses only the standard $C^{0}$ finite elements. Brenner and Sung [14] analyzed the $C^{0}$ interior penalty methods on polygonal domains using the Lagrange finite element. For a plate bending problem, in [15] we got an optimal estimate by the $C^{0}$ interior penalty method using Adini element and the penalty parameter was accurately estimated. Brenner et al. [16] developed isoparametric $C^{0}$ interior penalty methods on smooth domains and proved the optimal convergence in the energy norm. Comparing with the standard finite element method, the main advantages of the interior penalty method include the ability to capture discontinuities, and less restriction on grid structure and refinement as well as on the choice of basis functions.

In this paper, we use this idea and construct a semi-discrete scheme and a fully discrete scheme using the Wilson nonconforming element for the parabolic integro-differential equation arising in modeling the non-Fickian flow in porous media. Without using the conventional elliptic projection, which was an indispensable tool in the convergence analysis of finite element methods in previous literature, we get an optimal error estimate which is only determined by the interpolation error. Finally, we give some numerical experiments to show the efficiency of the method.

The rest of the paper is organized as follows. We give a semi-discrete scheme using the interior penalty method in Section 2. Section 3 contains the convergence analysis of the semi-scheme. In Section 4, we give the convergence analysis of the fully discrete scheme. Finally, some numerical experiments are carried out in Section 5.

## The semi-discrete scheme of non-Fickian flow in porous media

In this section, we give a new semi-discrete scheme using the interior penalty method. For simplicity, we consider the problem on a plane domain, that is, $\Omega\subset R^{2}$.

Suppose that $f=f(x,t)$ is a given smooth function, $A=A(x)$ and $B(t)=B(x,t)$ are $2\times2$ bounded matrices and *A* is strongly elliptic: there exist positive constants $k_{1},k_{2}$ and $a_{*},a^{*}$ such that

2.1 $$\begin{aligned} \begin{aligned} & 0< k_{1}\leq\phi\leq k_{2},\qquad 0< k_{1}\leq \Vert B \Vert \leq k_{2}, \qquad \biggl\Vert \frac{dB(t)}{dt} \biggr\Vert \leq k_{2} \\ & a_{*} \Vert \xi \Vert ^{2}\leq(A\xi,\xi)\leq a^{*} \Vert \xi \Vert ^{2},\quad \forall\xi\in R^{2}. \end{aligned} \end{aligned}$$

The variational form of (1.1) is to find $u:(0,T]\rightarrow H^{1}_{0}(\Omega)$, such that

2.2 $$ \textstyle\begin{cases}(\phi u_{t},v)+(A\nabla u,\nabla v) +\int_{0}^{t} (B(s)\nabla u(x,s),\nabla v )\,ds=(f,v),\quad \forall v \in H^{1}_{0}(\Omega),\\ u(x,0)=u_{0}(x),\quad \forall x\in\Omega. \end{cases} $$

Let $\{\mathcal{T}_{h}\}$ be a family of regular rectangle partitions of Ω. That is, denoted by $h_{T}, h$ the diameter of the element $T\in\mathcal{T}_{h}$ and $\max_{T\in\mathcal{T}_{h}} h_{T}$, and by $\rho_{T}$ the superior diameter of all circles contained in *T*, respectively, then it is assumed that $\frac{h_{T}}{\rho_{T}}\leq\sigma$ in which *σ* is a positive constant. We denote by $\{\mathcal{E}_{h}\}$ the set of all boundaries of $\mathcal{T}_{h}$. We write $h_{E}$ for the diameter of a boundary $E\in\mathcal{E}_{h}$.

Now introduce the jump and average of a piecewise smooth function *f* as follows. Let $E=\partial T\cap\partial T'$ be an interior boundary shared by two elements *T* and $T'$. Then the jump of *f* over *E* is defined by

$$[[f]]=f|_{T}-f|_{T'} $$

and the average as

$$\{f\}=\frac{1}{2}(f|_{T}+f|_{T'}). $$

The Wilson finite element is defined as follows. The freedom is described as

$$\Sigma_{T}= \biggl\{ v(a_{i}),1\leq i\leq4;\frac{h_{j}^{2}}{h_{1}h_{2}} \int_{T} \frac {\partial^{2}v}{\partial x_{j}^{2}}\,dx,j=1,2 \biggr\} . $$

The finite element space is defined as

$$\begin{aligned} V_{h}={}& \bigl\{ v_{h}:v_{h}|_{T}\in P_{2}(T);v_{h}|_{T}\mbox{ is uniquely determined by } \Sigma _{T}; \\ &v_{h}(a)=0\mbox{ for all nodes a on }\partial\Omega \bigr\} . \end{aligned}$$

$\Pi_{h}:H^{2}(\Omega)\rightarrow V_{h}$ is the corresponding interpolation operator and define that $(\cdot,\cdot)_{h}=\sum_{T\in\mathcal {T}_{h}} (\cdot,\cdot)_{T},\langle\cdot,\cdot\rangle_{h}=\sum_{E\in \mathcal {E}_{h}} \langle\cdot,\cdot\rangle_{E}$.

The traditional semi-discrete scheme is to find $u_{h}:(0,T]\rightarrow V_{h}$, such that

$$ \textstyle\begin{cases}(\phi u_{h,t},v_{h})_{h}+(A\nabla u_{h},\nabla v_{h})_{h} +\int_{0}^{t} (B(s)\nabla u_{h}(x,s),\nabla v_{h} )_{h}\, ds=(f,v_{h}),\quad \forall v_{h} \in V_{h},\\ u_{h}(x,0)=\Pi_{h}u_{0}(x),\quad \forall x\in\Omega. \end{cases} $$

The traditional norm in $V_{h}$ is defined as $\Vert v_{h} \Vert _{1,h}^{2}=\sum_{T} \vert v_{h} \vert _{1,T}^{2}$. The error estimation of this scheme is $\Vert u-u_{h} \Vert _{1,h}=O(h)$.

To improve the convergence order, we introduce a new semi-discrete scheme: Find $u_{h}:(0,T]\rightarrow V_{h}$, such that

2.3 $$ \textstyle\begin{cases}(\phi u_{h,t},v_{h})_{h}+a_{h}(u_{h},v_{h})+\int _{0}^{t}b_{h}(u_{h},v_{h})\,ds =(f,v_{h}), \quad \forall v_{h}\in V_{h},\\ u_{h}(x,0)=\Pi_{h}u_{0}(x),\quad \forall x\in\Omega, \end{cases} $$

in which

2.4 $$\begin{aligned} &a_{h}(u_{h},v_{h})=(A\nabla u_{h}, \nabla v_{h})_{h}+\sum_{E\in\mathcal {E}_{h}} \biggl\{ \frac{\alpha}{h_{E}} \bigl\langle [[u_{h}]], [[v_{h}]] \bigr\rangle _{E} \biggr\} \\ &\phantom{a_{h}(u_{h},v_{h})=} {}- \bigl\langle \{A\nabla u_{h}\}\cdot n, [[v_{h}]] \bigr\rangle _{h}- \bigl\langle [[u_{h}]],\{ A\nabla v_{h}\}\cdot n \bigr\rangle _{h}, \end{aligned}$$

2.5 $$\begin{aligned} &b_{h}(u_{h},v_{h})= \bigl(B(s)\nabla u_{h},\nabla v_{h} \bigr)_{h}+\sum _{E\in \mathcal {E}_{h}} \biggl\{ \frac{\alpha}{h_{E}} \bigl\langle [[u_{h}]], [[v_{h}]] \bigr\rangle _{E} \biggr\} \\ & \phantom{b_{h}(u_{h},v_{h})=} -\bigl\langle \bigl\{ B(s)\nabla u_{h} \bigr\} \cdot n, [[v_{h}]] \bigr\rangle _{h}- \bigl\langle [[u_{h}]], \bigl\{ B(s)\nabla v_{h} \bigr\} \cdot n \bigr\rangle _{h}, \end{aligned}$$

where *α* is a proper constant.

The new norm in the space $V_{h}$ is defined as

2.6 $$ \Vert v_{h} \Vert ^{2}_{h}=\sum _{T\in\mathcal{T}_{h}} \vert v_{h} \vert ^{2}_{1,T} +\sum_{E\in\mathcal{E}_{h}} \biggl\{ \frac{1}{h_{E}} \bigl\Vert [[v_{h}]] \bigr\Vert ^{2}_{0,E} \biggr\} , $$

which is larger than the traditional discrete norm.

To prove the convergence order of the new scheme, we first introduce several lemmas.

### Lemma 2.1

*There exists a positive constant* *C*, *such that*

$$\Vert w \Vert _{0,\partial T}^{2}\leq C \bigl(h_{T}^{-1} \Vert w \Vert ^{2}_{0,T}+h_{T} \vert w \vert ^{2}_{1,T} \bigr). $$

### Proof

$$\begin{aligned} \Vert w \Vert ^{2}_{0,\partial T} =& \int_{\partial T} \vert w \vert ^{2}\,d\tau= \int_{\partial\hat {T}} \vert \hat{w} \vert ^{2} \frac{ \vert \partial T \vert }{ \vert \partial\hat{T} \vert }\,d\hat{\tau}\leq Ch_{T}^{n-1} \Vert \hat{w} \Vert ^{2}_{0,\partial \hat{T}} \\ \leq&Ch_{T}^{n-1} \Vert \hat{w} \Vert ^{2}_{1,\hat {T}}=Ch_{T}^{n-1} \bigl( \Vert \hat{w} \Vert ^{2}_{0,\hat{T}}+ \vert \hat{w} \vert ^{2}_{1,\hat{T}} \bigr) \\ \leq&C \bigl(h_{T}^{-1} \Vert w \Vert ^{2}_{0,T}+h_{T} \vert w \vert ^{2}_{1,T} \bigr). \end{aligned}$$

The proof of the lemma is complete. □

### Lemma 2.2

*Let* $\{\mathcal{T}_{h}\}$ *be a regular rectangle partition of* Ω, *then there exists a positive constant* *C* *such that*

$$\begin{aligned} &h_{T} \Vert A\nabla v_{h}\cdot n \Vert ^{2}_{0,\partial T} \leq C \vert v_{h} \vert ^{2}_{1,T},\quad \forall v_{h}\in V_{h}, \\ &h_{T} \Vert B\nabla v_{h}\cdot n \Vert ^{2}_{0,\partial T} \leq C \vert v_{h} \vert ^{2}_{1,T}, \quad \forall v_{h}\in V_{h}. \end{aligned}$$

### Proof

By applying Lemma 2.1 and the inverse inequality, we have

$$\begin{aligned} \Vert A\nabla\cdot n \Vert ^{2}_{0,\partial T}&\leq C \bigl(h_{T}^{-1} \Vert A\nabla u_{h}\cdot n \Vert ^{2}_{0,T} +h_{T} \vert A\nabla u_{h}\cdot n \vert ^{2}_{1,T} \bigr) \\ &\leq C \bigl(h_{T}^{-1} \vert u_{h} \vert ^{2}_{1,T}+h_{T} \vert u_{h} \vert ^{2}_{2,T} \bigr) \\ &\leq C \biggl(h_{T}^{-1} \vert u_{h} \vert ^{2}_{1,T}+h_{T}\frac {1}{h_{T}^{2}} \vert u_{h} \vert ^{2}_{1,T} \biggr) \\ &\leq Ch_{T}^{-1} \vert u_{h} \vert ^{2}_{1,T}. \end{aligned}$$

The second inequality can be proved by the same argument. □

### Theorem 2.3

$a_{h}(\cdot,\cdot):V_{h}\times V_{h}\rightarrow\mathbb{R}$ *and* $b_{h}(\cdot ,\cdot):V_{h}\times V_{h}\rightarrow\mathbb{R}$ *are continuous and V*-*elliptic bilinear forms*.

### Proof

Obviously, they are bilinear forms.

According to definition (2.4), Hölder’s inequality and Lemma 2.2,

$$\begin{aligned} \bigl\vert a_{h}(u_{h},v_{h}) \bigr\vert \leq{}&\sum_{T\in\mathcal {T}_{h}}C \vert \nabla u_{h} \vert _{0,T} \vert \nabla v_{h} \vert _{0,T} + \sum_{E\in\mathcal{E}_{h}} \biggl\{ \frac{\alpha}{h_{E}} \bigl\Vert [[u_{h}]] \bigr\Vert _{0,E} \bigl\Vert [[v_{h}]] \bigr\Vert _{0,E} \\ &{} + \bigl\Vert \{A\nabla u_{h}\}\cdot n \bigr\Vert _{0,E} \bigl\Vert [[v_{h}]] \bigr\Vert _{0,E} + \bigl\Vert [[u_{h}]] \bigr\Vert _{0,E} \bigl\Vert \{A\nabla v_{h}\}\cdot n \bigr\Vert _{0,E} \biggr\} \\ \leq{}&\sum_{T\in\mathcal{T}_{h}}C \vert u_{h} \vert _{1,T} \vert v_{h} \vert _{1,T} +\sum _{E\in\mathcal{E}_{h}} \biggl\{ \frac{\alpha}{h_{E}} \bigl\Vert [[u_{h}]] \bigr\Vert _{0,E} \bigl\Vert [[v_{h}]] \bigr\Vert _{0,E} \\ &{} +\frac{C}{\sqrt{h_{E}}} \vert u_{h} \vert _{1,T} \bigl\Vert [[v_{h}]] \bigr\Vert _{0,E} + \frac{C}{\sqrt{h_{E}}} \bigl\Vert [[u_{h}]] \bigr\Vert _{0,E} \vert v_{h} \vert _{1,T} \biggr\} \\ \leq{}& C \biggl(\sum_{T\in\mathcal{T}_{h}} \vert u_{h} \vert _{1,T} +\sum_{E\in\mathcal{E}_{h}}\frac{1}{\sqrt{h_{E}}} \bigl\Vert [[u_{h}]] \bigr\Vert _{0,E} \biggr)\\ &{}\times\biggl(\sum_{T\in\mathcal{T}_{h}} \vert v_{h} \vert _{1,T} +\sum_{E\in\mathcal{E}_{h}}\frac{1}{\sqrt{h_{E}}} \bigl\Vert [[v_{h}]] \bigr\Vert _{0,E} \biggr)\\ \leq{}& C \Vert u_{h} \Vert _{h} \Vert v_{h} \Vert _{h}. \end{aligned}$$

So $a_{h}(\cdot,\cdot)$ is a continuous bilinear form.

Since

2.7 $$\begin{aligned} &\sum_{E\in\mathcal{E}_{h}} \int_{E} \bigl(\{A\nabla v_{h}\}\cdot n \bigr) [[v_{h}]]\, d\tau \\ &\quad\leq\sum_{E\in\mathcal{E}_{h}} \bigl\Vert \{A\nabla v_{h}\}\cdot n \bigr\Vert _{0,E} \bigl\Vert [[v_{h}]] \bigr\Vert _{0,E} \\ &\quad\leq \biggl(\sum_{E\in\mathcal{E}_{h}}h_{E} \bigl\Vert \{A\nabla v_{h}\} \cdot n \bigr\Vert ^{2}_{0,E} \biggr)^{\frac{1}{2}} \biggl(\sum_{E\in\mathcal{E}_{h}} \frac{1}{h_{E}} \bigl\Vert [[v_{h}]] \bigr\Vert ^{2}_{0,E} \biggr)^{\frac{1}{2}} \\ &\quad\leq C \biggl(\sum_{T\in\mathcal{T}_{h}}h_{T} \Vert A \nabla v_{h}\cdot n \Vert ^{2}_{0,\partial T} \biggr)^{\frac{1}{2}} \biggl(\sum_{E\in\mathcal{E}_{h}} \frac{1}{h_{E}} \bigl\Vert [[v_{h}]] \bigr\Vert ^{2}_{0,E} \biggr)^{\frac{1}{2}} \\ &\quad \leq C \biggl(\sum_{T\in\mathcal{T}_{h}} \vert v_{h} \vert ^{2}_{1,T} \biggr)^{\frac{1}{2}} \biggl(\sum _{E\in\mathcal{E}_{h}}\frac{1}{h_{E}} \bigl\Vert [[v_{h}]] \bigr\Vert ^{2}_{0,E} \biggr)^{\frac{1}{2}} \\ &\quad \leq\epsilon\sum_{T\in\mathcal{T}_{h}} \vert v_{h} \vert ^{2}_{1,T} +\frac{C}{4\epsilon}\sum _{E\in\mathcal{E}_{h}}\frac{1}{h_{E}} \bigl\Vert [[v_{h}]] \bigr\Vert ^{2}_{0,E}. \end{aligned}$$

Therefore,

$$\begin{aligned} &a_{h}(v_{h},v_{h}) \\ &\quad =\sum_{T\in\mathcal{T}_{h}} (A\nabla v_{h},\nabla v_{h})_{T} +\sum_{E\in\mathcal{E}_{h}} \biggl\{ \frac{\alpha }{h_{E}} \bigl\langle [[v_{h}]], [[v_{h}]] \bigr\rangle _{E} \biggr\} -2 \bigl\langle \{A\nabla v_{h}\}\cdot n, [[v_{h}]] \bigr\rangle _{h} \\ &\quad =\sum_{T\in\mathcal{T}_{h}} (A\nabla v_{h},\nabla v_{h})_{T} +\sum_{E\in\mathcal{E}_{h}} \biggl\{ \frac{\alpha}{h_{E}} \int _{E} [[v_{h}]] [[v_{h}]]\,d\tau -2 \int_{E} \bigl(\{A\nabla v_{h}\}\cdot n \bigr) [[v_{h}]]\,d\tau \biggr\} \\ &\quad= \sum_{T\in\mathcal{T}_{h}} \bigl\Vert A^{\frac{1}{2}}\nabla v_{h} \bigr\Vert ^{2}_{0,T} +\sum _{E\in\mathcal{E}_{h}}\frac{\alpha}{h_{E}} \bigl\Vert [[v_{h}]] \bigr\Vert ^{2}_{0,E} -\sum _{E\in\mathcal{E}_{h}}2 \int_{E} \bigl(\{A\nabla v_{h}\}\cdot n \bigr) [[v_{h}]]\, d\tau \\ &\quad \geq \sum_{T\in\mathcal{T}_{h}}a_{*} \vert v_{h} \vert ^{2}_{1,T} +\alpha\sum_{E\in\mathcal{E}_{h}} \frac{1}{h_{E}} \bigl\Vert [[v_{h}]] \bigr\Vert ^{2}_{0,E} -\epsilon\sum_{T\in\mathcal{T}_{h}} \vert v_{h} \vert ^{2}_{1,T} -\frac{C}{4\epsilon} \sum_{E\in\mathcal{E}_{h}}\frac{1}{h_{E}} \bigl\Vert [[v_{h}]] \bigr\Vert ^{2}_{0,E} \\ &\quad \geq \min \biggl\{ a_{*}-\epsilon,\alpha-\frac{C}{4\epsilon} \biggr\} \Vert v_{h} \Vert ^{2}_{h}. \end{aligned}$$

Select the appropriate value of *ϵ* independent of *h* to ensure $C_{1}=\min\{a_{*}-\epsilon,\alpha-\frac{C}{4\epsilon}\}>0$, then the bilinear form $a_{h}(\cdot,\cdot)$ is V-elliptic. By the same argument, with assumption (2.1), the bilinear form $b_{h}(\cdot,\cdot ):V_{h}\times V_{h}\rightarrow\mathbb{R}$ is also continuous and V-elliptic. Therefore the proof is complete. □

## Convergence of the new semi-discrete scheme

### Lemma 3.1

*Suppose* $u,u_{t}\in H^{3}(\Omega),u_{tt}\in H^{2}(\Omega)$ *and* $\Pi_{h}$ *is the interpolation operator*. *Then there holds*

$$\begin{aligned} &\bigl\Vert (u-\Pi_{h} u)_{tt} \bigr\Vert _{0} \leq Ch^{2} \vert u_{tt} \vert _{2},\qquad \Vert u- \Pi_{h} u \Vert _{h}\leq Ch^{2} \vert u \vert _{3}, \\ & \bigl\Vert (u-\Pi_{h} u)_{t} \bigr\Vert _{h}\leq Ch^{2} \vert u_{t} \vert _{3}. \end{aligned}$$

### Proof

The first inequality of the above conclusion is obvious according to the interpolation theory.

Since

$$\begin{aligned} \sum_{T\in\mathcal{T}_{h}} \vert u-\Pi_{h} u \vert ^{2}_{1,T}\leq Ch^{4} \vert u \vert ^{2}_{3} \end{aligned}$$

and

$$\begin{aligned} &\sum_{E\in\mathcal{E}_{h}} \biggl\{ \frac{1}{h_{E}} \bigl\Vert [[u-\Pi_{h} u]] \bigr\Vert ^{2}_{0,E} \biggr\} \\ &\quad \leq C\sum_{T\in\mathcal{T}_{h}} \biggl\{ \frac{1}{h_{T}} \Vert u-\Pi_{h} u \Vert ^{2}_{0,\partial T} \biggr\} \\ &\quad \leq C\sum_{T\in\mathcal{T}_{h}} \Vert \hat{u}-\widehat{\Pi _{h} u} \Vert ^{2}_{0,\partial\hat{T}} \leq C\sum _{T\in\mathcal{T}_{h}} \Vert \hat{u}-\widehat{\Pi_{h} u} \Vert ^{2}_{1,\hat {T}} \\ &\quad \leq C\sum_{T\in\mathcal{T}_{h}} \bigl\{ \Vert \hat{u}-\widehat{ \Pi _{h} u} \Vert ^{2}_{0,\hat{T}}+ \vert \hat{u} - \widehat{\Pi_{h} u} \vert ^{2}_{1,\hat{T}} \bigr\} \\ &\quad \leq C\sum_{T\in\mathcal{T}_{h}} \biggl\{ \frac{1}{h^{2}_{T}} \Vert u-\Pi _{h} u \Vert ^{2}_{0,T}+ \vert u- \Pi_{h} u \vert ^{2}_{1,T} \biggr\} \leq Ch^{4} \vert u \vert ^{2}_{3}. \end{aligned}$$

So

$$\begin{aligned} \Vert u-\Pi_{h} u \Vert ^{2}_{h}&=\sum _{T\in\mathcal {T}_{h}} \vert u-\Pi_{h} u \vert ^{2}_{1,T}+\sum_{E\in\mathcal{E}_{h}} \biggl\{ \frac{1}{h_{E}} \bigl\Vert [[u-\Pi_{h} u]] \bigr\Vert ^{2}_{0,E} \biggr\} \\ &\leq Ch^{4} \vert u \vert ^{2}_{3}. \end{aligned}$$

The second inequality $\Vert u-\Pi_{h} u \Vert _{h}\leq Ch^{2} \vert u \vert _{3}$ is obtained. The third inequality can be proved by the same argument. □

### Theorem 3.2

*Assume that* *u* *and* $u_{h}$ *are the solutions of* (2.2) *and* (2.3), *respectively*. *If* $u,u_{t}\in H^{3}(\Omega),u_{tt}\in H^{2}(\Omega)$, *then there exists a positive constant* *C* *such that*

$$\Vert u-u_{h} \Vert _{0}+ \int_{0}^{T} \Vert u-u_{h} \Vert _{h}\,ds \leq Ch^{2} \biggl( \vert u \vert _{2}+ \biggl[ \int_{0}^{T} \bigl( \vert u_{t} \vert _{2}^{2}+ \vert u \vert _{3}^{2} \bigr)\, dt \biggr]^{\frac{1}{2}} \biggr). $$

### Proof

Based on definition (2.4)-(2.5),

$$\begin{aligned} & a_{h}(u,v_{h})=(A\nabla u,\nabla v_{h})_{h}- \bigl\langle \{A\nabla u\cdot n\} , [[v_{h}]] \bigr\rangle _{h}, \\ & b_{h}(u,v_{h})= \bigl(B(t)\nabla u,\nabla v_{h} \bigr)_{h}- \bigl\langle \bigl\{ B(t)\nabla u\cdot n \bigr\} , [[v_{h}]] \bigr\rangle _{h}. \end{aligned}$$

Using the Green’s formula, we can get

$$\begin{aligned} &(\phi u_{t},v_{h})_{h}+a_{h}(u,v_{h})+ \int_{0}^{t}b_{h}(u,v_{h})\,ds \\ &\quad =(\phi u_{t},v_{h})- \bigl(\operatorname{div}(A\nabla u),v_{h} \bigr)- \int_{0}^{t} \bigl(\operatorname{div} \bigl(B(s) \nabla u \bigr),v_{h} \bigr)\,ds \\ &\quad =(\phi u_{t},v_{h})- \bigl(\operatorname{div}(A\nabla u),v_{h} \bigr)- \biggl(\operatorname{div} \biggl( \int_{0}^{t}B(s)\nabla u\,ds \biggr),v_{h} \biggr) \\ &\quad = \biggl(\phi u_{t}-\operatorname{div}(A\nabla u)-\operatorname {div} \biggl( \int_{0}^{t}B(s)\nabla u\,ds \biggr),v_{h} \biggr) \\ &\quad =(f,v_{h}), \quad \forall v_{h}\in V_{h}. \end{aligned}$$

Therefore,

$$\begin{aligned} & \bigl(\phi(u-u_{h})_{t},v_{h} \bigr)_{h}+a_{h}(u-u_{h},v_{h})+ \int _{0}^{t}b_{h}(u-u_{h},v_{h}) \,ds \\ &\quad =(\phi u_{t},v_{h})_{h}+a_{h}(u,v_{h})+ \int_{0}^{t}b_{h}(u,v_{h})\,ds \\ &\qquad{}- \biggl[(\phi u_{h,t},v_{h})_{h}+a_{h}(u_{h},v_{h}) + \int_{0}^{t}b_{h}(u_{h},v_{h}) \,ds \biggr] \\ &\quad = (f,v_{h})-(f,v_{h})=0. \end{aligned}$$

This is the key of the paper. Then we have

3.1 $$\begin{aligned} & \bigl(\phi(\Pi_{h} u-u_{h})_{t},v_{h} \bigr)_{h}+a_{h}(\Pi_{h} u-u_{h},v_{h})+ \int _{0}^{t}b_{h}(\Pi_{h}u-u_{h},v_{h}) \,ds \\ &\quad = \bigl(\phi(\Pi_{h} u-u)_{t},v_{h} \bigr)_{h}+a_{h}(\Pi_{h} u-u,v_{h})+ \int _{0}^{t}b_{h}(\Pi _{h}u-u,v_{h})\,ds, \quad \forall v_{h}\in V_{h}. \end{aligned}$$

Let $\theta_{h}=\Pi_{h} u-u_{h}$ and taking $v_{h}=\theta_{h}$ in (3.1), we can obtain

$$\begin{aligned} &\frac{k_{1}}{2}\frac{d}{dt} \Vert \theta_{h} \Vert _{0}^{2}+C \Vert \theta_{h} \Vert _{h}^{2} \\ &\quad \leq\frac{1}{2}\sum _{T\in\mathcal{T}_{h}}\frac{d}{dt} \bigl\Vert \phi^{\frac {1}{2}} \theta_{h} \bigr\Vert _{0,T}^{2} +a_{h}(\theta_{h},\theta_{h}) \\ &\quad = \bigl(\phi(\Pi_{h} u-u)_{t},\theta_{h} \bigr)_{h} +a_{h} \bigl((\Pi_{h} u-u), \theta_{h} \bigr)+ \int_{0}^{t}b_{h}(\Pi_{h}u-u, \theta _{h})\,ds \\ & \qquad{}- \int_{0}^{t} b_{h} \bigl( \theta_{h}(s),\theta_{h} \bigr)\,ds \\ &\quad \leq k_{2} \bigl\Vert (\Pi_{h} u-u)_{t} \bigr\Vert _{0} \Vert \theta _{h} \Vert _{0}+C \Vert \Pi_{h} u-u \Vert _{h} \Vert \theta _{h} \Vert _{h} \\ & \qquad{}+C \Vert \theta_{h} \Vert _{h} \int_{0}^{t} \bigl( \Vert \Pi_{h} u-u \Vert _{h}+ \bigl\Vert \theta_{h}(s) \bigr\Vert _{h} \bigr)\,ds \\ &\quad \leq Ch^{2} \vert u_{t} \vert _{2} \Vert \theta _{h} \Vert _{0}+Ch^{2} \vert u \vert _{3} \Vert \theta _{h} \Vert _{h} +C \Vert \theta_{h} \Vert _{h} \int_{0}^{t}h^{2} \vert u \vert _{3}\,ds \\ &\qquad{}+C \Vert \theta_{h} \Vert _{h} \int_{0}^{t} \bigl\Vert \theta _{h}(s) \bigr\Vert _{h}\,ds \\ &\quad \leq Ch^{4} \biggl(\frac{ \vert u_{t} \vert _{2}^{2}}{4\epsilon _{1}}+\frac{ \vert u \vert _{3}^{2}}{4\epsilon_{2}} + \int_{0}^{t}\frac{ \vert u \vert _{3}^{2}}{4\epsilon_{3}}\, ds \biggr)+ \epsilon_{1} \Vert \theta _{h} \Vert ^{2}_{0} +(\epsilon_{2}+\epsilon_{3}+ \epsilon_{4}) \Vert \theta _{h} \Vert ^{2}_{h} \\ & \qquad{}+\frac{1}{4\epsilon_{4}} \int_{0}^{t} \bigl\Vert \theta_{h}(s) \bigr\Vert ^{2}_{h}\,ds. \end{aligned}$$

Select the appropriate values of $\epsilon_{2}, \epsilon_{3}$ and $\epsilon _{4}$ independent of *h* to ensure

3.2 $$ \frac{d}{dt} \Vert \theta_{h} \Vert _{0}^{2}+ \Vert \theta_{h} \Vert _{h}^{2}\leq Ch^{4} \biggl( \vert u_{t} \vert _{2}^{2}+ \vert u \vert _{3}^{2}+ \int_{0}^{t} \vert u \vert _{3}^{2} \,ds \biggr) +C \biggl( \Vert \theta_{h} \Vert ^{2}_{0} + \int_{0}^{t} \bigl\Vert \theta_{h}(s) \bigr\Vert ^{2}_{h}\,ds \biggr). $$

Integrating both sides of (3.2) from 0 to *T* and noticing that $\theta_{h}(0)=0$, we obtain

$$\begin{aligned} \Vert \theta_{h} \Vert _{0}^{2}+ \int_{0}^{T} \Vert \theta _{h} \Vert _{h}^{2}\,ds \leq{}&Ch^{4} \int_{0}^{T} \bigl( \vert u_{t} \vert _{2}^{2}+ \vert u \vert _{3}^{2} \bigr)\,ds \\ & {}+C \int_{0}^{T} \biggl( \Vert \theta_{h} \Vert ^{2}_{0}+ \int _{0}^{t} \bigl\Vert \theta_{h}(s) \bigr\Vert ^{2}_{h}\,ds \biggr)\,dt. \end{aligned}$$

Gronwall’s lemma now implies

$$\begin{aligned} \Vert \theta_{h} \Vert _{0}^{2}+ \int_{0}^{T} \Vert \theta _{h} \Vert _{h}^{2}\,ds \leq Ch^{4} \int_{0}^{T} \bigl( \vert u_{t} \vert _{2}^{2}+ \vert u \vert _{3}^{2} \bigr)\,ds. \end{aligned}$$

So the error between the discrete solution $u_{h}$ and the exact solution *u* is

3.3 $$\begin{aligned} \Vert u-u_{h} \Vert _{0}+ \int_{0}^{T} \Vert u-u_{h} \Vert _{h}\,ds \leq Ch^{2} \biggl( \vert u \vert _{2}+ \biggl[ \int_{0}^{T} \bigl( \vert u_{t} \vert _{2}^{2}+ \vert u \vert _{3}^{2} \bigr)\, dt \biggr]^{\frac{1}{2}} \biggr). \end{aligned}$$

The proof of the theorem is complete. □

## Analysis for the fully discrete scheme

Denote by $\Delta t=T/N$ the time increment, where *N* is a positive integer,

$$t_{n}=n\Delta t,\qquad u^{n}=u(\cdot,t_{n}),\qquad D_{t}u^{n}= \frac{u^{n}-u^{n-1}}{\Delta t},\quad n=1,2,\ldots,N. $$

For a given smooth function $f(s)$, we have that

$$\int_{t_{i-1}}^{t_{i}}f(s)\,ds=\Delta tf(t_{i})+ \epsilon_{i}(f),\qquad \Biggl\Vert \sum_{i=1}^{n} \epsilon_{i}(f) \Biggr\Vert =O(\Delta t). $$

Then the fully discrete scheme can be formulated as follows:

For $n=1,2,\ldots,N$, find $u_{h}^{n}\in V_{h}$ such that

4.1 $$\begin{aligned} \textstyle\begin{cases}(\phi D_{t}u_{h}^{n},v_{h})_{h}+a_{h}(u_{h}^{n},v_{h}) +\Delta t\sum_{i=1}^{n} b_{h}(u_{h}^{i},v_{h})=(f^{n},v_{h}),\quad \forall v_{h}\in V_{h},\\ u_{h}^{0}=\Pi_{h} u^{0}, \end{cases}\displaystyle \end{aligned}$$

in which

4.2 $$\begin{aligned} &a_{h} \bigl(u_{h}^{n},v_{h} \bigr)= \bigl(A\nabla u_{h}^{n},\nabla v_{h} \bigr)_{h} +\sum_{E\in\mathcal{E}_{h}} \biggl\{ \frac{\alpha}{h_{E}} \int _{E} \bigl[ \bigl[u_{h}^{n} \bigr]\bigr] [[v_{h}]]\,ds \biggr\} \\ &\phantom{a_{h} \bigl(u_{h}^{n},v_{h} \bigr)=}{}- \bigl\langle \bigl\{ A\nabla u_{h}^{n} \bigr\} \cdot n, [[v_{h}]] \bigr\rangle _{h} - \bigl\langle \bigl[ \bigl[u_{h}^{n} \bigr]\bigr],\{A\nabla v_{h}\}\cdot n \bigr\rangle _{h}, \end{aligned}$$

4.3 $$\begin{aligned} &b_{h} \bigl(u_{h}^{i},v_{h} \bigr)= \bigl(B^{i}\nabla u_{h}^{i},\nabla v_{h} \bigr)_{h} +\sum_{E\in\mathcal{E}_{h}} \biggl\{ \frac{\alpha}{h_{E}} \int _{E} \bigl[ \bigl[u_{h}^{i} \bigr]\bigr] [[v_{h}]]\,ds \biggr\} \\ &\phantom{b_{h} \bigl(u_{h}^{i},v_{h} \bigr)=}{}- \bigl\langle \bigl\{ B^{i}\nabla u_{h}^{i} \bigr\} \cdot n, [[v_{h}]] \bigr\rangle _{h} - \bigl\langle \bigl[ \bigl[u_{h}^{i} \bigr]\bigr], \bigl\{ B^{i}\nabla v_{h} \bigr\} \cdot n \bigr\rangle _{h}. \end{aligned}$$

### Theorem 4.1

*The fully discrete scheme* (4.1) *has one and only one solution*.

### Proof

Let $\{\varphi_{i}\}^{r}_{i=1}$ be a set of basis functions in $V_{h}$, then $u^{n}_{h}$ can be expressed as $u^{n}_{h}=\sum_{i=1}^{r} \psi_{i}(t_{n})\varphi_{i}$. Select $v_{h}=\varphi _{j}\ (j=1,\ldots,r)$ and scheme (4.1) can be written as follows: Find $\psi _{i}(t_{n})\ (i=1,\ldots,r)$, such that

4.4 $$\begin{aligned} \textstyle\begin{cases} (X+\Delta tY+(\Delta t)^{2}Z^{n} )\psi(t_{n})\\ \quad = X\psi(t_{n-1})-(\Delta t)^{2}\sum_{i=1}^{n-1} Z^{n-1}\psi (t_{i})+\Delta tF,\quad \forall v_{h}\in V_{h},\\ \psi(0)=\psi_{0}, \end{cases}\displaystyle \end{aligned}$$

where $\psi_{0}$ is given by $u_{h}^{0}=\Pi_{h}u^{0}=\sum_{i=1}^{r} \psi _{i}(0)\varphi_{i}$, and

$$\begin{aligned} &X= \bigl(\phi(\varphi_{i},\varphi_{j}) \bigr)_{r\times r},\qquad Y=(Y_{ij})_{r\times r},Z^{n}= \bigl(Z^{n}_{ij} \bigr)_{r\times r}, \\ &F= \bigl((f,\varphi_{i}) \bigr)_{1\times r},\qquad \psi(t_{n})= \bigl(\psi_{1}(t_{n}),\cdot, \psi_{r}(t_{n}) \bigr)^{T}, \\ &Y_{ij}=(A\nabla\varphi_{i},\nabla\varphi_{j})_{h} +\sum_{E} \biggl\{ \frac{\alpha}{h_{E}} \bigl\langle [[\varphi_{i}]], [[\varphi _{j}]] \bigr\rangle _{E} \biggr\} \\ &\phantom{Y_{ij}=}{}- \bigl\langle \{A\nabla\varphi_{i}\}\cdot n, [[ \varphi_{j}]] \bigr\rangle _{h} - \bigl\langle [[ \varphi_{i}]],\{A\nabla\varphi_{j}\}\cdot n \bigr\rangle _{h}, \\ &Z^{n}_{ij}= \bigl(B^{n}\nabla \varphi_{i},\nabla\varphi_{j} \bigr)_{h} +\sum _{E} \biggl\{ \frac{\alpha}{h_{E}} \bigl\langle [[ \varphi_{i}]], [[\varphi _{j}]] \bigr\rangle _{E} \biggr\} \\ &\phantom{Z^{n}_{ij}=} - \bigl\langle \bigl\{ B^{n}\nabla\varphi_{i} \bigr\} \cdot n, [[\varphi_{j}]] \bigr\rangle _{h} - \bigl\langle [[\varphi_{i}]], \bigl\{ B^{n}\nabla \varphi_{j} \bigr\} \cdot n \bigr\rangle _{h}. \end{aligned}$$

The coefficient matrix $X+\Delta tY+(\Delta t)^{2}Z^{n}$ is a symmetric positive definite matrix, so scheme (4.1) has one and only one solution. □

### Theorem 4.2

*Assume that* $u^{n}$ *and* $u^{n}_{h}$ *are the solutions of* (2.2) *and* (4.1), *respectively*. *If* $u^{n},u^{n}_{t}\in H^{3}(\Omega),u^{n}_{tt}\in H^{2}(\Omega)$, *then there exists a positive constant* *C* *such that*

$$\begin{aligned} \bigl\Vert u^{n}-u_{h}^{n} \bigr\Vert _{h} \leq{}& Ch^{2} \bigl\vert u^{n} \bigr\vert _{3}+Ch^{2} \Biggl(\sum_{i=1}^{n} \bigl( \bigl\vert u_{t}^{i} \bigr\vert ^{2}_{2}+ \bigl\vert u_{t}^{i} \bigr\vert ^{2}_{3}+ \bigl\vert u^{i} \bigr\vert ^{2}_{3} \bigr) \Biggr)^{\frac{1}{2}} \\ &{} +C\Delta t \Biggl(\sum_{i=1}^{n} \bigl( \bigl\Vert u_{tt}^{i} \bigr\Vert ^{2}_{0}+ \bigl\vert u^{i} \bigr\vert ^{2}_{2} \bigr) \Biggr)^{\frac{1}{2}}. \end{aligned}$$

### Proof

Based on definition (4.2)-(4.3),

$$\begin{aligned} a_{h} \bigl(u^{n},v_{h} \bigr)= \bigl(A\nabla u^{n},\nabla v_{h} \bigr)_{h}- \bigl\langle \bigl\{ A\nabla u^{n}\cdot n \bigr\} , [[v_{h}]] \bigr\rangle _{h}, \\ b_{h} \bigl(u^{i},v_{h} \bigr)= \bigl(B^{i}\nabla u^{i},\nabla v_{h} \bigr)_{h} - \bigl\langle \bigl\{ B^{i}\nabla u^{i} \cdot n \bigr\} , [[v_{h}]] \bigr\rangle _{h}. \end{aligned}$$

Using the Green’s formula, we can get

$$\begin{aligned} & \bigl(\phi D_{t}u^{n},v_{h} \bigr)_{h}+a_{h} \bigl(u^{n},v_{h} \bigr)+\Delta t\sum_{i=1}^{n} b_{h} \bigl(u^{i},v_{h} \bigr) \\ &\quad = \bigl(\phi D_{t}u^{n},v_{h} \bigr)- \bigl( \operatorname{div} \bigl(A\nabla u^{n} \bigr),v_{h} \bigr) - \Delta t\sum_{i=1}^{n} \bigl( \operatorname{div} \bigl(B^{i}\nabla u^{i} \bigr),v_{h} \bigr) \\ &\quad = \bigl(\phi u_{t}^{n},v_{h} \bigr)- \bigl( \operatorname{div} \bigl(A\nabla u^{n} \bigr),v_{h} \bigr) - \biggl( \int_{0}^{t_{n}}\operatorname{div} \bigl(B(s)\nabla u \bigr)\, ds,v_{h} \biggr) + \bigl(R_{1}^{n}+R_{2}^{n},v_{h} \bigr) \\ &\quad = \biggl(\phi u_{t}^{n}-\operatorname{div} \bigl(A\nabla u^{n} \bigr)-\operatorname {div} \biggl( \int _{0}^{t_{n}}B(s)\nabla u\,ds \biggr),v_{h} \biggr) + \bigl(R_{1}^{n}+R_{2}^{n},v_{h} \bigr) \\ &\quad = \bigl(f^{n},v_{h} \bigr)+ \bigl(R_{1}^{n}+R_{2}^{n},v_{h} \bigr),\quad \forall v_{h}\in V_{h}, \end{aligned}$$

in which

4.5 $$\begin{aligned} &R_{1}^{n}=\phi \bigl(D_{t}u^{n}-u_{t}^{n} \bigr)=O(\Delta t), \end{aligned}$$

4.6 $$\begin{aligned} &R_{2}^{n}= \int_{0}^{t_{n}}\operatorname{div} \bigl(B(s)\nabla u \bigr)\,ds -\Delta t\sum_{i=1}^{n} \operatorname{div} \bigl(B^{i}\nabla u^{i} \bigr)=O(\Delta t). \end{aligned}$$

Set $u^{n}-u_{h}^{n}=u^{n}-\Pi_{h}u^{n}+\Pi_{h}u^{n}-u_{h}^{n}=\eta^{n}+\theta^{n}$. There holds the following error equation:

4.7 $$\begin{aligned} &\bigl(\phi D_{t}\theta^{n},v_{h} \bigr)_{h}+a_{h} \bigl(\theta^{n},v_{h} \bigr)+\Delta t\sum_{i=1}^{n} b_{h} \bigl(\theta^{i},v_{h} \bigr) \\ &\quad = \bigl(-\phi D_{t}\eta^{n},v_{h} \bigr)_{h}-a_{h} \bigl(\eta^{n},v_{h} \bigr)-\Delta t\sum_{i=1}^{n} b_{h} \bigl(\eta^{i},v_{h} \bigr) + \bigl(R_{1}^{n}+R_{2}^{n},v_{h} \bigr),\quad\forall v_{h}\in V_{h}. \end{aligned}$$

Take $v_{h}=\theta^{n}$ in (4.7), we can obtain

$$\begin{aligned} &\frac{k_{1}}{2\Delta t} \bigl( \bigl\Vert \theta^{n} \bigr\Vert _{0}^{2}- \bigl\Vert \theta^{n-1} \bigr\Vert _{0}^{2} \bigr)+C \bigl\Vert \theta^{n} \bigr\Vert _{h}^{2}\\ &\quad \leq \bigl(\phi D_{t} \theta^{n},\theta^{n} \bigr)_{h}+a_{h} \bigl(\theta^{n},\theta ^{n} \bigr) \\ &\quad = \bigl(-\phi D_{t}\eta^{n},\theta^{n} \bigr)_{h}-a_{h} \bigl(\eta^{n}, \theta^{n} \bigr)-\Delta t\sum_{i=1}^{n} b_{h} \bigl(\eta^{i},\theta^{n} \bigr) -\Delta t \sum_{i=1}^{n} b_{h} \bigl( \theta^{i},\theta ^{n} \bigr)+ \bigl(R_{1}^{n}+R_{2}^{n}, \theta ^{n} \bigr) \\ &\quad \leq k_{2} \bigl\Vert D_{t} \eta^{n} \bigr\Vert _{0} \bigl\Vert \theta ^{n} \bigr\Vert _{0}+C \bigl\Vert \eta^{n} \bigr\Vert _{h} \bigl\Vert \theta^{n} \bigr\Vert _{h}+ C \Delta t \Biggl(\sum_{i=1}^{n} \bigl\Vert \eta^{i} \bigr\Vert _{h} \Biggr) \bigl\Vert \theta^{n} \bigr\Vert _{h} \\ &\qquad{}+C\Delta t \Biggl(\sum _{i=1}^{n} \bigl\Vert \theta^{i} \bigr\Vert _{h} \Biggr) \bigl\Vert \theta^{n} \bigr\Vert _{h} \\ &\qquad {}+C \bigl\Vert R_{1}^{n} \bigr\Vert _{0} \bigl\Vert \theta^{n} \bigr\Vert _{0}+C \bigl\Vert R_{2}^{n} \bigr\Vert _{0} \bigl\Vert \theta^{n} \bigr\Vert _{0}. \end{aligned}$$

That is,

4.8 $$\begin{aligned} &\frac{k_{1}}{2} \bigl( \bigl\Vert \theta^{n} \bigr\Vert _{0}^{2}- \bigl\Vert \theta^{n-1} \bigr\Vert _{0}^{2} \bigr)+C\Delta t \bigl\Vert \theta^{n} \bigr\Vert _{h}^{2} \\ &\quad \leq k_{2}\Delta t \bigl\Vert D_{t}\eta^{n} \bigr\Vert _{0} \bigl\Vert \theta^{n} \bigr\Vert _{0}+C\Delta t \bigl\Vert \eta ^{n} \bigr\Vert _{h} \bigl\Vert \theta^{n} \bigr\Vert _{h}+ C(\Delta t)^{2} \Biggl(\sum_{i=1}^{n} \bigl\Vert \eta^{i} \bigr\Vert _{h} \Biggr) \bigl\Vert \theta^{n} \bigr\Vert _{h} \\ &\qquad{}+C(\Delta t)^{2} \Biggl(\sum _{i=1}^{n} \bigl\Vert \theta^{i} \bigr\Vert _{h} \Biggr) \bigl\Vert \theta^{n} \bigr\Vert _{h} +C\Delta t \bigl\Vert R_{1}^{n} \bigr\Vert _{0} \bigl\Vert \theta^{n} \bigr\Vert _{0}+C\Delta t \bigl\Vert R_{2}^{n} \bigr\Vert _{0} \bigl\Vert \theta ^{n} \bigr\Vert _{0}. \end{aligned}$$

Now we analyze the right-hand side of (4.8) by *ε*-Cauchy inequality.

4.9 $$\begin{aligned} &k_{2}\Delta t \bigl\Vert D_{t}\eta^{n} \bigr\Vert _{0} \bigl\Vert \theta ^{n} \bigr\Vert _{0}=k_{2} \biggl\Vert \int _{t_{n-1}}^{t_{n}}(I-\Pi_{h})u_{t} \,dt \biggr\Vert _{0} \bigl\Vert \theta ^{n} \bigr\Vert _{0} \\ &\phantom{k_{2}\Delta t \bigl\Vert D_{t}\eta^{n} \bigr\Vert _{0} \bigl\Vert \theta ^{n} \bigr\Vert _{0}}\leq C\Delta th^{4} \int_{t_{n-1}}^{t_{n}} \vert u_{t} \vert _{2}^{2}\, dt+\epsilon_{1} \bigl\Vert \theta^{n} \bigr\Vert ^{2}_{0}, \end{aligned}$$

4.10 $$\begin{aligned} &C\Delta t \bigl\Vert \eta^{n} \bigr\Vert _{h} \bigl\Vert \theta ^{n} \bigr\Vert _{h} \leq C\Delta th^{2} \bigl\vert u^{n} \bigr\vert _{3} \bigl\Vert \theta ^{n} \bigr\Vert _{h} \leq C\Delta th^{4} \bigl\vert u^{n} \bigr\vert ^{2}_{3}+\epsilon_{2}\Delta t \bigl\Vert \theta^{n} \bigr\Vert ^{2}_{h}, \end{aligned}$$

4.11 $$\begin{aligned} &C(\Delta t)^{2}\sum_{i=1}^{n} \bigl\Vert \eta^{i} \bigr\Vert _{h} \bigl\Vert \theta^{n} \bigr\Vert _{h} \leq C(\Delta t)^{2}h^{2}\sum_{i=1}^{n} \bigl\vert u^{i} \bigr\vert _{3} \bigl\Vert \theta^{n} \bigr\Vert _{h} \\ &\phantom{C(\Delta t)^{2}\sum_{i=1}^{n} \bigl\Vert \eta^{i} \bigr\Vert _{h} \bigl\Vert \theta^{n} \bigr\Vert _{h}}\leq C(\Delta t)^{3}h^{4}\sum_{i=1}^{n} \bigl\vert u^{i} \bigr\vert ^{2}_{3}+ \epsilon_{3}\Delta t \bigl\Vert \theta^{n} \bigr\Vert ^{2}_{h}, \end{aligned}$$

4.12 $$\begin{aligned} &C(\Delta t)^{2} \Biggl(\sum _{i=1}^{n} \bigl\Vert \theta^{i} \bigr\Vert _{h} \Biggr) \bigl\Vert \theta^{n} \bigr\Vert _{h} \leq C(\Delta t)^{3}\sum_{i=1}^{n} \bigl\Vert \theta^{i} \bigr\Vert ^{2}_{h}+ \epsilon_{4}\Delta t \bigl\Vert \theta^{n} \bigr\Vert ^{2}_{h}. \end{aligned}$$

According to the definition of $R_{1}^{n}$ and *ε*-Cauchy inequality, we get

4.13 $$\begin{aligned} &C\Delta t \bigl\Vert R_{1}^{n} \bigr\Vert _{0} \bigl\Vert \theta^{n} \bigr\Vert _{0} \\ &\quad=C \biggl\Vert \int _{t_{j-1}}^{t_{j}}(t_{j-1}-t)u_{tt} \,dt \biggr\Vert _{0} \bigl\Vert \theta ^{n} \bigr\Vert _{0} \\ &\quad \leq C\Delta t \int_{t_{j-1}}^{t_{j}} \Vert u_{tt} \Vert _{0}\, dt \bigl\Vert \theta^{n} \bigr\Vert _{0} \leq C(\Delta t)^{3} \int_{t_{j-1}}^{t_{j}} \Vert u_{tt} \Vert ^{2}_{0}\,dt+\epsilon_{5} \bigl\Vert \theta^{n} \bigr\Vert ^{2}_{0}, \end{aligned}$$

4.14 $$\begin{aligned} &C\Delta t \bigl\Vert R_{2}^{n} \bigr\Vert _{0} \bigl\Vert \theta^{n} \bigr\Vert _{0} \\ &\quad = C \Delta t \Biggl\Vert \sum_{i=1}^{n} \int_{t_{i-1}}^{t_{i}} \bigl[\operatorname{div} \bigl(B(s) \nabla u \bigr)-\operatorname {div} \bigl(B^{i}\nabla u^{i} \bigr) \bigr]\,dt \Biggr\Vert _{0} \bigl\Vert \theta^{n} \bigr\Vert _{0} \\ &\quad \leq C(\Delta t)^{2}\sum_{i=1}^{n} \bigl\vert u^{i}_{t} \bigr\vert _{2} \bigl\Vert \theta^{n} \bigr\Vert _{0} \leq C(\Delta t)^{4}\sum_{i=1}^{n} \bigl\vert u^{i}_{t} \bigr\vert ^{2}_{2}+ \epsilon_{6} \bigl\Vert \theta^{n} \bigr\Vert ^{2}_{0}. \end{aligned}$$

Combining the above inequalities from (4.8) to (4.14), and choosing the $\{\epsilon_{i}\}_{i=2}^{4}$ small enough, we can obtain

4.15 $$\begin{aligned} &\frac{k_{1}}{2} \bigl( \bigl\Vert \theta^{n} \bigr\Vert _{0}^{2}- \bigl\Vert \theta^{n-1} \bigr\Vert _{0}^{2} \bigr)+C\Delta t \bigl\Vert \theta^{n} \bigr\Vert _{h}^{2} \\ &\quad \leq C\Delta th^{4} \int_{t_{n-1}}^{t_{n}} \vert u_{t} \vert _{2}^{2}\,dt+C\Delta th^{4} \bigl\vert u^{n} \bigr\vert ^{2}_{3} +C(\Delta t)^{3}h^{4}\sum_{i=1}^{n} \bigl\vert u^{i} \bigr\vert ^{2}_{3}+C(\Delta t)^{3} \int _{t_{j-1}}^{t_{j}} \Vert u_{tt} \Vert ^{2}_{0}\,dt \\ &\qquad{}+C(\Delta t)^{4}\sum_{i=1}^{n} \bigl\vert u^{i}_{t} \bigr\vert ^{2}_{2} +(\epsilon_{1}+\epsilon_{5}+\epsilon_{6}) \bigl\Vert \theta^{n} \bigr\Vert ^{2}_{0} +C(\Delta t)^{3}\sum_{i=1}^{n} \bigl\Vert \theta^{i} \bigr\Vert ^{2}_{h}. \end{aligned}$$

Sum up from $i=1$ to *N*, applying Gronwall’s inequality and noticing that $\theta(0)=0$, we can get

4.16 $$\begin{aligned} & \bigl\Vert \theta^{N} \bigr\Vert _{0}^{2}+C \Delta t\sum_{i=1}^{N} \bigl\Vert \theta^{i} \bigr\Vert _{h}^{2} \\ &\quad \leq C\Delta th^{4} \int_{0}^{T} \vert u_{t} \vert _{2}^{2}\, dt+C\Delta th^{4}\sum _{i=1}^{N} \bigl\vert u^{i} \bigr\vert ^{2}_{3} +C(\Delta t)^{3} \int_{0}^{T} \Vert u_{tt} \Vert ^{2}_{0}\, dt \\ &\qquad{} +C(\Delta t)^{3}\sum_{i=1}^{N} \bigl\vert u^{i}_{t} \bigr\vert ^{2}_{2}. \end{aligned}$$

So we get

4.17 $$\begin{aligned} \sum_{i=1}^{N} \bigl\Vert \theta^{i} \bigr\Vert _{h}^{2} \leq{}& Ch^{4} \int_{0}^{T} \vert u_{t} \vert _{2}^{2}\,dt+Ch^{4}\sum _{i=1}^{N} \bigl\vert u^{i} \bigr\vert ^{2}_{3} +C(\Delta t)^{2} \int_{0}^{T} \Vert u_{tt} \Vert ^{2}_{0}\, dt \\ &{}+C(\Delta t)^{2}\sum_{i=1}^{N} \bigl\vert u^{i}_{t} \bigr\vert ^{2}_{2}. \end{aligned}$$

Therefore,

4.18 $$\begin{aligned} \bigl\Vert \theta^{n} \bigr\Vert _{h}&\leq \Biggl(\sum _{i=1}^{N} \bigl\Vert \theta^{i} \bigr\Vert _{h}^{2} \Biggr)^{\frac{1}{2}} \\ &\leq Ch^{2} \Biggl( \int_{0}^{T} \vert u_{t} \vert _{2}^{2}\, dt+\sum_{i=1}^{N} \bigl\vert u^{i} \bigr\vert ^{2}_{3} \Biggr)^{\frac{1}{2}} +C\Delta t \Biggl( \int_{0}^{T} \Vert u_{tt} \Vert ^{2}_{0}\,dt +\sum_{i=1}^{N} \bigl\vert u^{i}_{t} \bigr\vert ^{2}_{2} \Biggr)^{\frac{1}{2}}. \end{aligned}$$

So

$$\begin{aligned} \bigl\Vert u^{n}-u_{h}^{n} \bigr\Vert _{h} \leq{}& Ch^{2} \bigl\vert u^{n} \bigr\vert _{3}+Ch^{2} \Biggl( \int_{0}^{T} \vert u_{t} \vert _{2}^{2}\,dt+\sum_{i=1}^{N} \bigl\vert u^{i} \bigr\vert ^{2}_{3} \Biggr)^{\frac{1}{2}} \\ &{}+C\Delta t \Biggl( \int_{0}^{T} \Vert u_{tt} \Vert ^{2}_{0}\,dt +\sum_{i=1}^{N} \bigl\vert u^{i}_{t} \bigr\vert ^{2}_{2} \Biggr)^{\frac{1}{2}}. \end{aligned}$$

 □

## Numerical example

Consider the parabolic integro-differential boundary value problem:

$$\begin{aligned} \textstyle\begin{cases} u_{t}-\Delta u-\int_{0}^{t}\Delta u(x,s)\,ds = ((1+4\pi^{2})e^{t}-2\pi^{2} )\sin(\pi x)\sin(\pi y),& \mbox{in }\Omega \times(0,T],\\ u=0,&\mbox{on }\partial\Omega\times(0,T],\\ u(x,0)=\sin(\pi x)\sin(\pi y),& \forall x\in\Omega, \end{cases}\displaystyle \end{aligned}$$

in which $\Omega=[0,1]\times[0,1]$ and $T=1s$. The real solution of this equation is $u= e^{t}\sin(\pi x)\sin(\pi y)$. Assume that the time interval $[0,1]$ is divided into *M* uniform subintervals by point $0=t^{0}< t^{1}< t^{2}<\cdots<t^{M}=1$, where $t^{n}=n\triangle t$. Moreover, define $u^{n}=u(\cdot,n\triangle t)$ for $0\leq n\leq M$ and denote the first-order backward Euler difference quotient as $u_{t}(\cdot ,n\triangle t)=\frac{u^{n+1}-u^{n}}{\triangle t}$.

Then the fully discrete scheme can be formulated as follows:

For $n=1,2,\ldots,M$, find $u_{h}^{n}\in V_{h}$ such that

5.1 $$\begin{aligned} \textstyle\begin{cases}(\frac{u_{h}^{n}-u_{h}^{n-1}}{\Delta t},v_{h})_{h}+a_{h}(u_{h}^{n},v_{h}) +\Delta t\sum_{i=1}^{n} b_{h}(u_{h}^{i},v_{h})=(f^{n},v_{h}),\quad \forall v_{h}\in V_{h},\\ u_{h}^{0}=\Pi_{h} u^{0}, \end{cases}\displaystyle \end{aligned}$$

in which

5.2 $$\begin{aligned} & a_{h} \bigl(u_{h}^{n},v_{h} \bigr)= \bigl(A\nabla u_{h}^{n},\nabla v_{h} \bigr)_{h} +\sum_{E\in\mathcal{E}_{h}} \biggl\{ \frac{\alpha}{h_{E}} \int _{E} \bigl[ \bigl[u_{h}^{n} \bigr]\bigr] [[v_{h}]]\,ds \biggr\} \\ &\phantom{a_{h} \bigl(u_{h}^{n},v_{h} \bigr)=}{}- \bigl\langle \bigl\{ A\nabla u_{h}^{n} \bigr\} \cdot n, [[v_{h}]] \bigr\rangle _{h} - \bigl\langle \bigl[ \bigl[u_{h}^{n} \bigr]\bigr],\{A\nabla v_{h}\}\cdot n \bigr\rangle _{h}, \end{aligned}$$

5.3 $$\begin{aligned} &b_{h} \bigl(u_{h}^{i},v_{h} \bigr)= \bigl(B^{i}\nabla u_{h}^{i},\nabla v_{h} \bigr)_{h} +\sum_{E\in\mathcal{E}_{h}} \biggl\{ \frac{\alpha}{h_{E}} \int _{E} \bigl[ \bigl[u_{h}^{i} \bigr]\bigr] [[v_{h}]]\,ds \biggr\} \\ &\phantom{b_{h} \bigl(u_{h}^{i},v_{h} \bigr)=}- \bigl\langle \bigl\{ B^{i}\nabla u_{h}^{i} \bigr\} \cdot n, [[v_{h}]] \bigr\rangle _{h} - \bigl\langle \bigl[ \bigl[u_{h}^{i} \bigr]\bigr], \bigl\{ B^{i}\nabla v_{h} \bigr\} \cdot n \bigr\rangle _{h}. \end{aligned}$$

Select $\alpha=6$ and $\Delta t=\frac{1}{2N^{2}}s$, in which $N^{2}$ is the square partition of Ω, we respectively get the error and the order at $t=0.4s,0.7s,1s$ in Table 1.

**Table 1:** **The error and order at** $\pmb{t=0.4s,0.7s,1.0s}$ **, respectively.**

$\boldsymbol {N^{2}} $	**Error**	**Order**	**Error**	**Order**	**Error**	**Order**
2 × 2	1.0844	-	1.5356	-	1.8760	-
4 × 4	0.3886	1.4805	0.4826	1.6699	0.6084	1.6246
8 × 8	0.0937	2.0522	0.1327	1.8627	0.1607	1.9207
16 × 16	0.0260	1.8495	0.0319	2.0565	0.0397	2.0172
32 × 32	0.0058	2.1644	0.0082	1.9599	0.0098	2.0183

**Figure 1:**
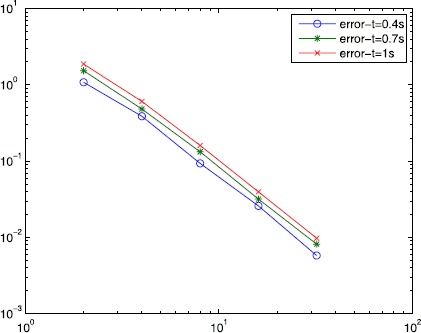
**The error estimate at** ***t*** **=0.4, 0.7s and 1s.**

The curve of the error estimate at $t=0.4s,0.7s,1.0s$ is drawn in Figure 1.

The following graphics describe the discrete solution $u_{h}$ and the real solution *u* at $t=1s$, respectively.

**Figure 2:**
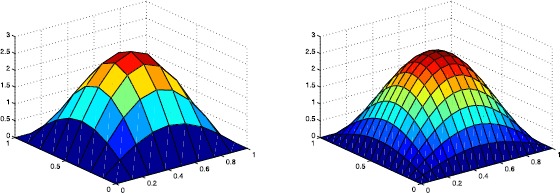
**The surfaces of** $\pmb{u_{h}}$ **at** $\pmb{t=1s}$ **when** $\pmb{h=\frac{1}{8}}$ **and** $\pmb{h=\frac{1}{16}}$ **, respectively.**

**Figure 3:**
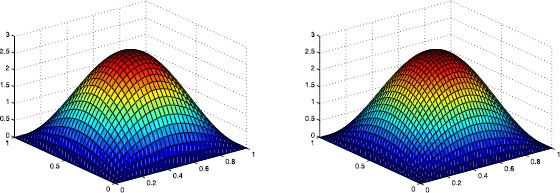
**The surfaces of** $\pmb{u_{h}}$ **and** ***u*** **at** $\pmb{t=1s}$ **when** $\pmb{h=\frac {1}{32}}$ **, respectively.**

From Table 1 and Figures 2 and 3, we can see that with the increase in the number of meshes, the discrete solution $u_{h}$ approximates to the real solution *u*. The convergence of scheme (5.1) using Wilson element is approximative of order $O(h^{2})$ from the table and Figure 1. Therefore, the numerical result is consistent with the theoretical analysis.

## Conclusions

In this paper, for the parabolic integro-differential equation, we present a new nonconforming scheme in which the consistency term vanishes. Therefore, we get an optimal error estimate which is only determined by the interpolation error. Finally, some numerical experiments show the efficiency of the method.
